# Detection of Rifampicin Resistance in *Mycobacterium tuberculosis* by Padlock Probes and Magnetic Nanobead-Based Readout

**DOI:** 10.1371/journal.pone.0062015

**Published:** 2013-04-22

**Authors:** Anna Engström, Teresa Zardán Gómez de la Torre, Maria Strømme, Mats Nilsson, David Herthnek

**Affiliations:** 1 Department of Microbiology, Tumor and Cell Biology, Karolinska Institutet, Stockholm, Sweden; 2 Department of Preparedness, Swedish Institute for Communicable Disease Control, Solna, Sweden; 3 Department of Engineering Sciences, Division of Nanotechnology and Functional Materials, Uppsala University, The Ångström Laboratory, Uppsala, Sweden; 4 Science for Life Laboratory, Department of Biochemistry and Biophysics, Stockholm University, Stockholm, Sweden; 5 Department of Immunology, Genetics and Pathology, Uppsala University, Science for Life Laboratory, Rudbeck Laboratory, Uppsala, Sweden; St. Petersburg Pasteur Institute, Russian Federation

## Abstract

Control of the global epidemic tuberculosis is severely hampered by the emergence of drug-resistant *Mycobacterium tuberculosis* strains. Molecular methods offer a more rapid means of characterizing resistant strains than phenotypic drug susceptibility testing. We have developed a molecular method for detection of rifampicin-resistant *M. tuberculosis* based on padlock probes and magnetic nanobeads. Padlock probes were designed to target the most common mutations associated with rifampicin resistance in *M. tuberculosis*, i.e. at codons 516, 526 and 531 in the gene *rpoB*. For detection of the wild type sequence at all three codons simultaneously, a padlock probe and two gap-fill oligonucleotides were used in a novel assay configuration, requiring three ligation events for circularization. The assay also includes a probe for identification of the *M. tuberculosis* complex. Circularized probes were amplified by rolling circle amplification. Amplification products were coupled to oligonucleotide-conjugated magnetic nanobeads and detected by measuring the frequency-dependent magnetic response of the beads using a portable AC susceptometer.

## Introduction

Tuberculosis (TB), caused by *Mycobacterium tuberculosis*, remains as a major public health problem. Increasing resistance to anti-TB drugs severely threatens the control of the disease. Prompt detection of drug-resistant *M. tuberculosis* strains is crucial for prescription of appropriate treatment. However, conventional drug susceptibility testing is a very time-consuming procedure, requiring weeks to months to complete due to the slow growth of the causative agent. In order to quickly detect drug-resistant *M. tuberculosis* it is therefore essential to use molecular based diagnostic methods, which can be performed within a day.

Chromosomal mutations are the genetic basis for drug resistance in *M. tuberculosis* [1], [2]. The effective first line anti-TB drug rifampicin (RIF) inhibits transcription by binding to the ß-subunit (encoded by *rpoB*) of the RNA polymerase [3]. Resistance to RIF in *M. tuberculosis* is almost entirely associated with mutations within an 81-bp region of the *rpoB* gene, called the RIF resistance-determining region (RRDR), comprising codons 507 to 533 [2], [4]. A substitution in the first or second nucleotide position of codons 516 and 526 or in the second nucleotide position in codon 531 are the most commonly observed mutations in RIF-resistant clinical isolates of *M. tuberculosis* [5].

Padlock probes are linear oligonucleotides that comprise two target-specific sequences at the 3′ and 5′ ends, and a linker segment containing sequences for amplification and detection [6]. The padlock probe ends are brought into juxtaposition upon hybridization to the target sequence, allowing padlock probe circularization by ligation. A mismatch near the ligation junction, and in particular at the 3′ end of the probe, prevents ligation [7], providing a specific means of mutation detection. Padlock probes were chosen due to this promising virtue of specificity and ease of multiplexing [8], [9], [10],[11], while the various strategies to improve the specificity of allele-specific PCR are often insufficient, elaborate to design and can lead to compromised amplification efficiency [12], [13]. Isothermal signal amplification of the padlock probe is achieved through rolling circle amplification (RCA) of the reacted padlock probes [14], [15]. The concatemer of replicated padlock probe produced by RCA is restriction digested, re-ligated into new circles and subjected to an additional round of RCA, known as circle-to-circle amplification (C2CA) [8], increasing the sensitivity of the assay.

Nanomedicine is just starting to reshape clinical practice [16] and since last decade magnetic nanoparticles, or so called nanobeads, have drawn increasing attention to the development of different types of magnetic biosensors. This is because of their high physical and chemical stability and that they are not generally affected by reagent chemistry or exposure to light. Magnetic nanobead-based biosensors offer an attractive and cost-effective route for detection of biomolecules, since they are relatively inexpensive to produce and easily made biocompatible [17]. The portable readout instrument for the detection principle described below should be possible to produce inexpensively, as it contains no optics but only electromagnetic device components. The Brownian relaxation principle constitutes a substrate-free biosensor method, where suspended magnetic nanobeads exhibiting Brownian relaxation behavior [18] are equipped with probe molecules for recognition of specific target molecules. Hybridization of target molecules to the probes causes a hydrodynamic size increase of the nanobeads, corresponding to the size of the target molecule. This brings on a decrease in the relaxation frequency of the beads, defined by the position of the peak in the imaginary part 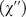 of the complex magnetization spectrum 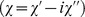, since the frequency is inversely proportional to the hydrodynamic size of the beads. The concentration of the targets can be monitored as a corresponding decrease of the amplitude of the relaxation peak 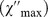 of the free beads [19], and several targets can, in principle, be monitored simultaneously by employing differently sized beads [20]. This strategy is employed in the volume amplified magnetic nanobead detection assay (VAM-NDA) [19], [21], [22], [23].

We have developed a molecular method for detection of RIF resistance in *M. tuberculosis* by padlocks probes, RCA and a magnetic nanobead-based readout; VAM-NDA. The nine most common *rpoB* mutations, located in codons 516, 526 and 531, were targeted by a cocktail of padlock probes, making use of their high suitability for multiplexing [8]. The assay includes a probe for species detection and a novel type of padlock probe system confirming loss of wild type at any of the three investigated RRDR codons.

## Materials and Methods

### Bacteria, DNA Extraction and Fragmentation

The reference strains *M. tuberculosis* H37Rv (ATCC 25618), *M. avium* (ATCC 25291), *M. marinum* (ATCC 2275), *M. microti* (ATCC 19422), and clinical isolates *M. interjectum* s99/96, *M. kansasaii* Alk Prague, *M. szulgai* BTB 98-526, *M. canetti* BTB 04-106, *M. bovis* BTB 08-329, *M. africanum* XTB 08-066 were included in the study. In addition, eight RIF-resistant *M. tuberculosis* clinical isolates, each harboring a 516 TAC, 516 GTC, 526 CGC, 526 CTC, 526 AAC, 526 TAC, 531 TTG or 531 TGG *rpoB* mutation, were also included [24]. Bacteria were cultured on Löwenstein-Jensen (LJ) medium or on LJ-medium containing 40 mg/L RIF prior to DNA extraction, as previously described [25]. Ten micrograms of genomic DNA was fragmented by 10 U each of NaeI and HpyCH4V (New England Biolabs, Ipswich, MA, USA) at 37°C for 90 min, following enzyme inactivation at 65°C for 20 min. DNA concentration was measured by Qubit dsDNA HS and BR assays (Invitrogen, Carlsbad, CA, USA).

### Padlock Probes and Oligonucleotides

Sequences were obtained from the *M. tuberculosis* H37Rv genome (GenBank accession no. NC_000962; NCBI bank) [26] and the Tuberculosis Drug Resistance Mutation Database [5]. Sequences of padlock probes and oligonucleotides used in the study (Integrated DNA Technologies, Inc.,Coralville, IA, USA) are specified in in the supporting information. A wild type padlock probe system consisting of a padlock probe and two gap-fill oligonucleotides (P5875, L11420, L11170) was designed to hybridize to *rpoB* codons 511 to 534 with ligation sites at the first nucleotide positions of codons 516 and 526, and the second nucleotide position of codon 531. Nine mutant-specific padlock probes were designed for detection of mutations at the first and second nucleotide positions of *rpoB* codons 516 and 526, and at the second nucleotide position of *rpoB* codon 531. Mutant-specific probes targeting the same nucleotide position but different nucleotides were ordered degenerated at the 3′ end nucleotide position of the probe (526 CKC, 526 DAC and 531 TKG). A padlock probe was designed for the 16S–23S internal transcribed spacer (ITS) region for detection of the *M. tuberculosis* complex (MTC). The hybridizing padlock probe arms were designed to have salt adjusted melting temperatures (Tm) between 50°C and 60°C, as calculated by the online software OligoCalc version 3.26 [27]. The backbone of the padlock probes linking the arms together consisted of hybridization sites for restriction oligonucleotides [28] and detection oligonucleotides. Due to possible coincidental matching of backbone with arms or disposition for folding due to high GC content [29], predictions of secondary structures were made using the online tool Mfold Web Server [30] with default settings. Efforts were made to avoid structures with low free energy (ΔG) having high probability of forming [31], by adjusting the lengths of the arms.

Padlock probes and the gap-fill oligonucleotides L11420, L11170 and L11171 were phosphorylated at the 5′ end by mixing 1 µM oligonucleotide with 1× PNK buffer A, 1 mM ATP (Fermentas, Vilnius, Lithuania), and 1 U/µl T4 polynucleotide kinase (Fermentas), and incubating at 37°C for 30 min followed by enzyme inactivation at 65°C for 20 min. The phosphorylated oligonucleotides were stored at −20°C until use.

### Padlock Probe Ligation and Amplification

Target recognition and amplification by C2CA was performed essentially as previously described [32]. Three nanogram of genomic DNA were used for specificity testing of the wild type probe system, the mutant-specific padlock probes, and the *M. tuberculosis* complex padlock probe. Thirty nanogram of genomic DNA were used for the final test of the complete assay. One microliter of template DNA (synthetic or genomic) was added to a 19 µl ligation mixture consisting of 1× ligase buffer (see below), 250 mU/µl Ampligase (EpiCenter, Madison, WI, USA) or Tth DNA ligase (GeneCraft, Cologne, Germany), 0.2 µg/µl Bovine Serum Albumin (BSA, New England Biolabs), 33 nM of each phosphorylated padlock probe, or 100 nM of phosphorylated P4782, P4949, or P5875, and 50 nM of the capture probe. The gap-fill oligonucleotides were added in 50 nM to the wild type probe system reactions. When Ampligase was applied, the ligase buffer comprised of 18 mM TRIS-HCl pH 8.3, 22.5 mM KCl (Merck, Whitehouse Station, NJ, USA), 9 mM MgCl_2_ (Sigma-Aldrich, St. Loius, MO, USA), 0.009% Triton X-100 (Sigma-Aldrich) and 0.5 mM NAD (Sigma-Aldrich), and when GeneCraft’s Tth DNA ligase was applied, the included reaction buffer was used. Genomic DNA was denaturated at 95°C for 4 min prior to use. Hybridization and ligation were performed at 60°C for 5 min. MyOne Dynabeads T1 (Invitrogen) were washed three times in 1× Wtw buffer (10 mM TRIS-HCl pH 7.5, 5 mM EDTA, 0.1% Tween 20 [Sigma-Aldrich], 0.1 M NaCl). Unreacted padlock probes were removed by capture of the target DNA to 50 µg Dynabeads by coupling to the biotinylated capture oligonucleotide followed by a wash with 1× Wtw buffer using a permanent magnet. Twenty microliters of RCA mixture containing 0.2 µg/µl BSA, 125 µM dNTP (Fermentas), 1× Phi29 buffer (Fermentas) and 100 mU/µl Phi29 DNA polymerase (Fermentas) was added to the Dynabeads. RCA was performed at 37°C for 20 min followed by enzyme inactivation at 65°C for 1 min. To monomerize the RCA products, restriction oligonucleotide L12890 (120 nM) was added with 0.2 µg/µl BSA and 120 mU/µl of *Alu*I (New England Biolabs) in 1× Phi29 buffer followed by incubation at 37°C for 1 min and enzyme inactivation at 65°C for 1 min. After the beads were discarded, the monomerized RCA products were re-circularized and amplified by addition of 1× Phi29 buffer, 0.2 µg/µl BSA, 100 µM dNTP, 0.68 mM ATP, 60 mU/µl Phi29 DNA polymerase, 14 mU/µl T4 DNA ligase (Fermentas) and incubated at 37°C for 20 min followed by enzyme inactivation at 65°C for 1 min.

### Improvements of the Wild Type Probe System

Initial probe design was compared to improved oligonucleotides by applying different sets of wild type probe components on 10 amol synthetic target DNA (L10919). The padlock probe and the longer gap-fill oligonucleotide were stepwise exchanged to the improved versions (P4782 to P4949, and L11171 to L11420). For concordance with the detection oligonucleotide used by the other probes, P4949 was subsequently modified to P5875.

### Digital Quantification of RCA Products

RCA products were quantified using a single-molecule detection method [33] (herein referred to as SMD), as briefly described below. A fluorescent dye-coupled oligonucleotide (L13179, 5 nM, except for the wild type probe system improvements test described above where L10806 was used) was hybridized to the RCA products in a reaction mixture consisting of 20 mM EDTA, 20 mM TRIS-HCl, 0.1% Tween 20 and 1 M NaCl, for 2 min at 70°C and 15 min at 55°C. The sample was pumped through a microchannel mounted on a standard confocal fluorescence microscope operating in a line-scan mode, and individual RCA products were detected and quantified in Matlab 7.0 (MathWorks, MA, USA).

### Coupling of Oligonucleotides to Magnetic Nanobeads

Two hundred microliters of spherical avidin-functionalized magnetic nanobeads (Micromod Partikeltechnologie GmbH, Rostock, Germany) with a nominal bead diameter of 100 nm was washed twice with 1× Wtw buffer using a permanent magnet. The beads were thereafter resuspended in 100 µl 1× Wtw buffer and incubated with 20 µl of 10 µM biotin-conjugated oligonucleotides (L9261) for 30 min at room temperature. The beads were washed twice and resuspended in 200 µl 1× PBS (pH 7.5).

### Magnetic Bead-based Readout

After dilution of the oligonucleotide-tagged beads to a concentration of 2 mg/ml, 20 µl was added to 100 µl of RCA products. The solution was incubated for 20 min at 60°C and thereafter diluted with 80 µl of a mixture containing equal volume of 1× PBS (pH 7.5) and a solution containing 20 mM EDTA, 20 mM TRIS-HCl, 0.1% Tween 20 and 1 M NaCl. Measurement of the frequency-dependent magnetic response at 24°C was performed in a DynoMag®-instrument (Acreo, Sweden, frequency range 1 Hz–100 kHz and AC field amplitude 0.5 mT). It should be noted that there were unavoidable variations in magnetic material in each sample. Therefore, in order to normalize the magnetic response with respect to the amount of magnetic material in each sample, the data was normalized against the constant value of the in-phase component of the volume susceptibility (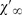) as this value is proportional to the total content of iron-oxide nanoparticles in a sample as previously described [23]. The 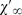 was measured at frequencies well above the Brownian relaxation frequency. In the current work, the limit of detection (LOD) was defined as the lowest tested amount of DNA yielding a normalized magnetic response differing more than three standard deviations of the negative control.

## Results

**Figure 1:**
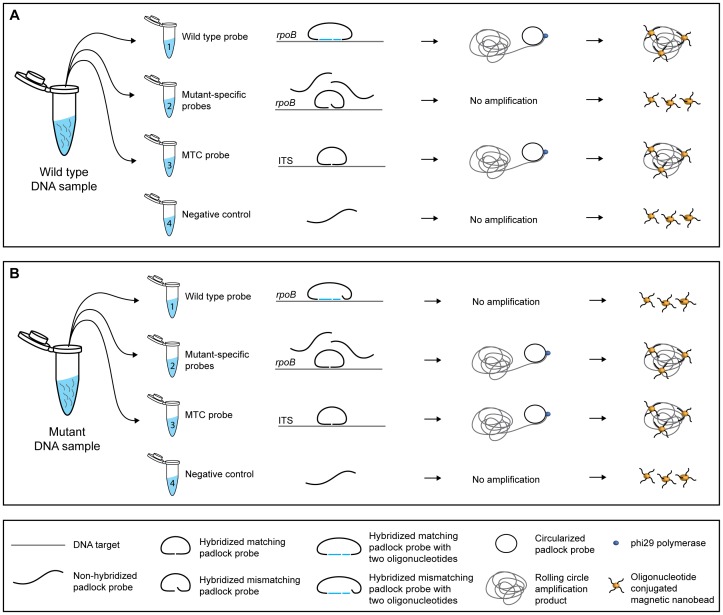
Outline of the molecular method. A DNA sample is distributed into three tubes containing (1) the wild type padlock probe system (a padlock probe and two gap-fill oligonucleotides with ligation sites at *rpoB* codons 516, 526 and 531), (2) a cocktail of nine mutant-specific padlock probes for detection of mutations at codons 516, 526 and 531, and (3) an *M. tuberculosis* complex (MTC)-specific padlock probe targeting the 16S–23S intergenic spacer region (ITS). For each probe system, there was also a (4) template negative control. After hybridization and ligation of the padlock probes, the circularized probes are amplified by rolling circle amplification (RCA). Oligonucleotide-conjugated magnetic nanobeads are added to each tube, and the frequency-dependent magnetic response is measured. The RCA products are detected by the decrease in the Brownian relaxation magnetic susceptibility peak of non-coupled beads. A: a wild type *rpoB* DNA sample yields signal only in tube 1 and tube 3. B: a DNA sample with mutation in *rpoB* codon 531 yields signal only in tube 2 and 3.

A molecular method for detection of RIF-resistant *M. tuberculosis* was developed. The method is outlined in Figure 1 and consists of a novel wild type padlock probe system comprising a padlock probe and two gap-fill oligonucleotides, a cocktail of nine mutant-specific padlock probes for detection of mutations in *rpoB* codons 516, 526 and 531, and an *M. tuberculosis* complex (MTC)-specific padlock probe. Efficiency, specificity and multiplexing of probes were investigated by the SMD method, and the complete assay was read out by the VAM-NDA using a portable AC susceptometer.

### Development of the Wild Type Probe System

In order to confirm loss of wild type due to presence of any of the mutations detected by the mutation-specific padlocks, a novel design for analyzing several codons simultaneously was developed. A padlock probe was designed to hybridize upstream of codon 516 and downstream of codon 531, with its 3′ end targeting specifically the second nucleotide position of codon 531, the most commonly mutated nucleotide in RIF-resistant *M. tuberculosis* [2], [5]. In between the padlock probe arms, two gap-filling oligonucleotides were designed with their 3′ ends targeting the first nucleotide of codons 516 and 526. For the wild type probe system to be able to circularize in spite of the long rigid duplex [34] formed by the three target-hybridizing oligonucleotides, the originally 38 nucleotides long backbone was extended by poly-T spacers to 48 nucleotides while the padlock arms were shortened, reducing the duplex length from 80 to 72 nucleotides. To relieve strain in the duplex further, the longest gap-fill oligonucleotide (between codons 516 and 526) was designed to contain one deletion and two deliberate target mismatches to make it act as a hinge. Both these measures increased the efficacy of the system significantly (Figure S1). Each of the improved probe system components alone increased the product yield by more than 10 times. Together, they increased the signal more than 60 times.

### Specificity Testing of Wild Type Probe System

**Figure 2:**
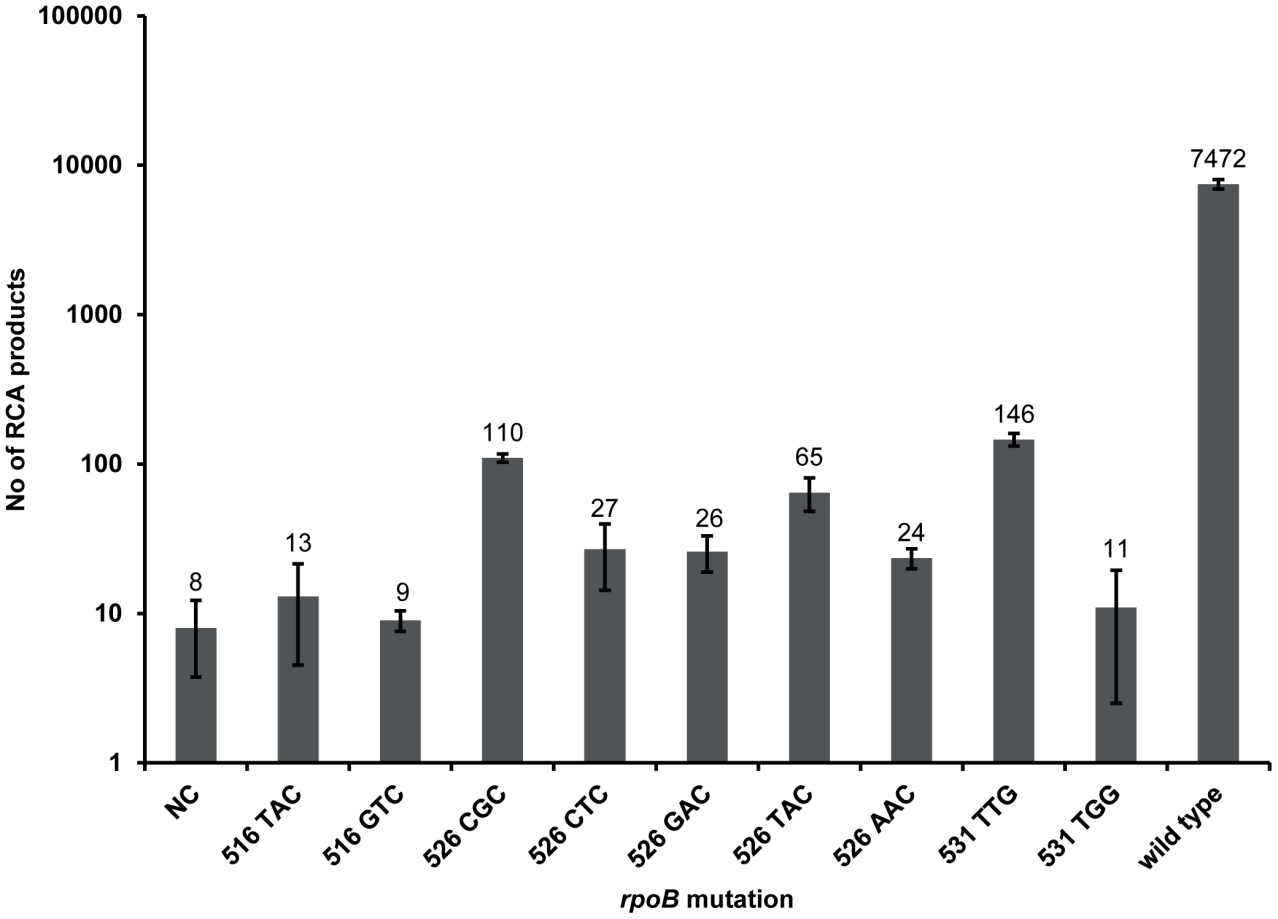
Specificity testing of wild type probe system. The system was tested on the reference strain *M. tuberculosis* H37Rv (representing wild type *rpoB*), eight *M. tuberculosis* clinical isolates, each harboring an rpoB 516 TAC, 516 GTC, 526 CGC, 526 CTC, 526 TAC, 526 AAC, 531 TTG or 531 TGG mutation, and a synthetic target representing an *rpoB* 526 GAC mutation. RCA products were hybridized to fluorescent dye-coupled oligonucleotide and visualized in a confocal microscope. NC: negative control.

Specificity of the wild type probe system was evaluated on DNA samples from a collection of *M. tuberculosis* strains. These include the RIF-susceptible reference strain *M. tuberculosis* H37Rv, harboring a wild type *rpoB* gene, and eight RIF-resistant *M. tuberculosis* clinical isolates, harboring a mutation at the first or second nucleotide position of *rpoB* codons 516 and 526, or a mutation at the second nucleotide position of *rpoB* codon 531. The ratios between the signals of matching (wild type *rpoB*) and mismatching (mutant *rpoB*) target ranged from 51 (*rpoB* 531 TTG mutation) to 830 (*rpoB* 516 GTC mutation) (Figure 2), demonstrating robust discrimination between the wild type and mutant strains.

### Testing of Specificity and Multiplexability of Mutant-specific Padlock Probes

**Figure 3:**
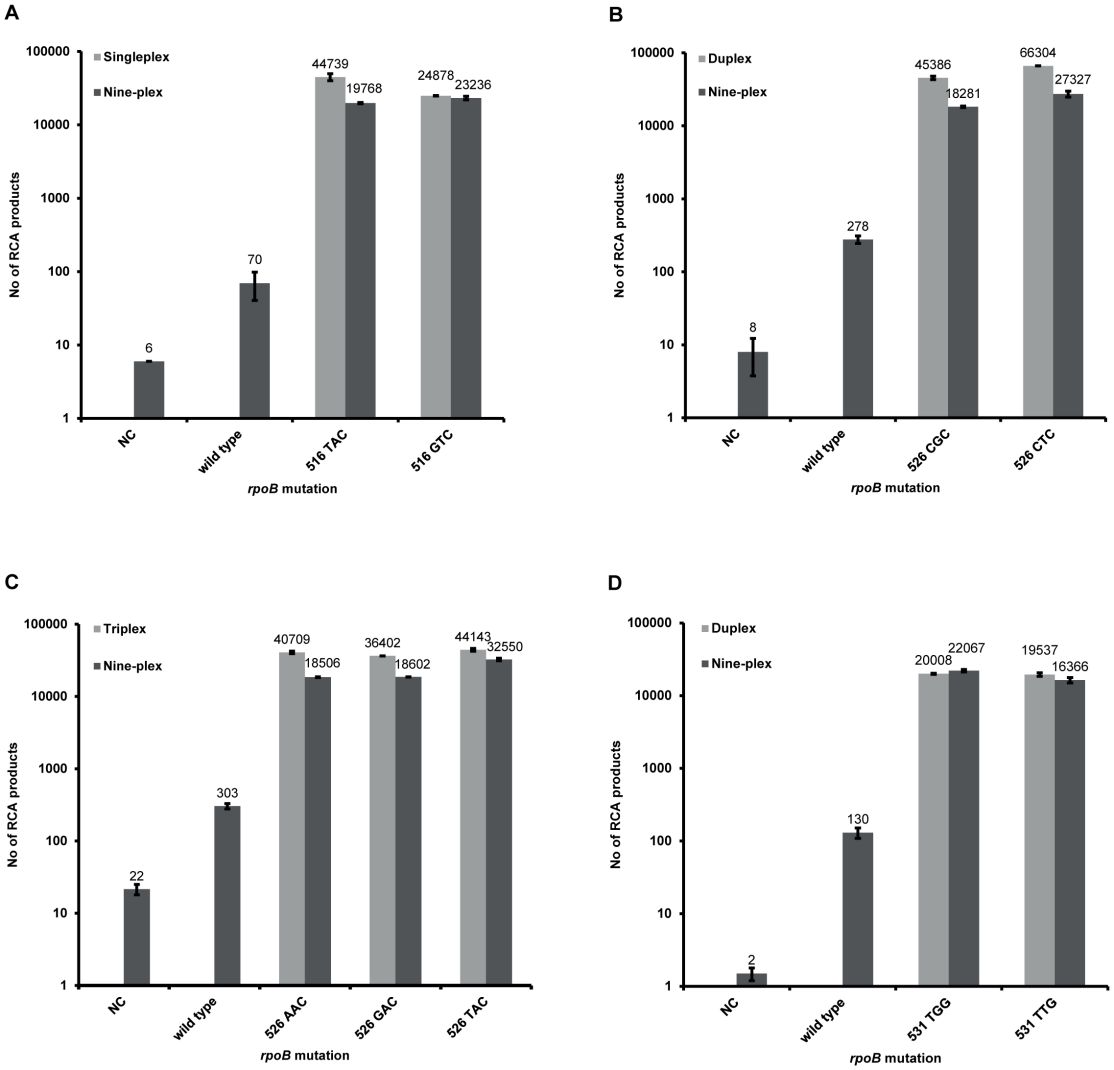
Specificity and multiplexability testing of the mutant-specific padlock probes. The probes were tested in single-, du-, or tri-plex and nine-plex (all mutant-specific padlock probes) on eight *M. tuberculosis* clinical isolates harboring an *rpoB* 516 TAC or GTC mutation (A), an *rpoB* 526 CGC or 526 CTC mutation (B), an *rpoB* 526 AAC or 526 TAC mutation (C), an *rpoB* 531 TTG or 531 TGG mutation (D), on a synthetic target with an *rpoB* 526 GAC mutation (C), and on the reference strain *M. tuberculosis* H37Rv (representing wild type *rpoB*) (A–D). RCA products were hybridized to fluorescent dye-coupled oligonucleotide and visualized in a confocal microscope. NC: negative control.

Specificity and multiplexability of the nine mutant-specific padlock probes were tested on their respective matching target (mutant *rpoB*) and on mismatching target (wild type *rpoB*) (Figure 3). All probes were assessed to be specific, with a matching/mismatching signal ratio ranging from 61 (526 AAC probe; Figure 3C) to 332 (516 GTC probe; Figure 3A). Efficiency of probes was compared between single-, du-, or triplex reactions to the full nine-plex assay. The mutant-specific padlock probes that were designed to target the same nucleotide position but different nucleotides were synthesized degenerated at the 3′ end nucleotide of the probe (526 CKC, 526 DAC and 531 TKG), thus these probes could not be evaluated in a singleplex format, but were instead evaluated in a duplex (526 CKC and 531 TKG) or triplex (526 DAC) format. The efficiency reduction of nine-plex reactions (Figure 3) was marginal (maximum 2-fold signal decrease) and all probes yielded a comparable signal on their respective matching target.

### Specificity Testing of the *M. tuberculosis* Complex Probe

**Figure 4:**
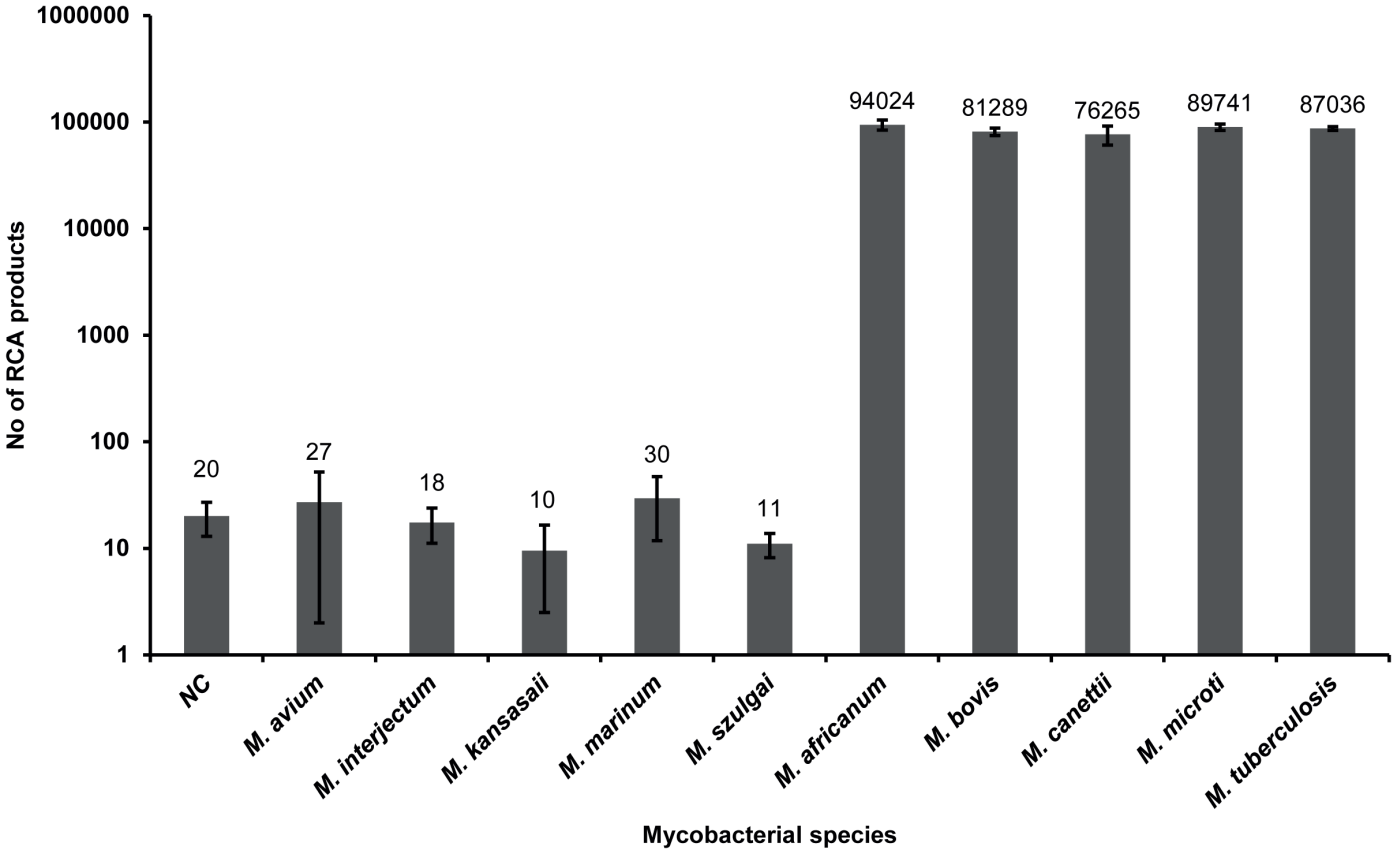
Specificity testing of the *M. tuberculosis* complex padlock probe. The probe, designed to target the 16S–23S intergenic spacer region, was tested on five *M. tuberculosis* complex species; *M. africanum*, *M. bovis*, *M. canetti*, *M. microti* and *M. tuberculosis*, and on five nontuberculous mycobacterial species; *M. avium*, *M. interjectum*, *M. kansasaii*, *M. marinum*, *M. szulgai*. RCA products were hybridized to fluorescent dye-coupled oligonucleotide and visualized in a confocal microscope. NC: negative control.

The *M. tuberculosis* complex padlock probe targeting the 16S–23S ITS region proved to be specific when tested on a range of mycobacterial species, including five members of the *M. tuberculosis* complex; *M. africanum*, *M. bovis*, *M. canetti*, *M. microti* and *M. tuberculosis*, and five nontuberculous mycobacterial species; *M. avium*, *M. interjectum*, *M. kansasaii*, *M. marinum*, *M. szulgai* (Figure 4). The matching/mismatching signal ratio was greater than 2500.

### Magnetic Bead-based Readout

**Figure 5:**
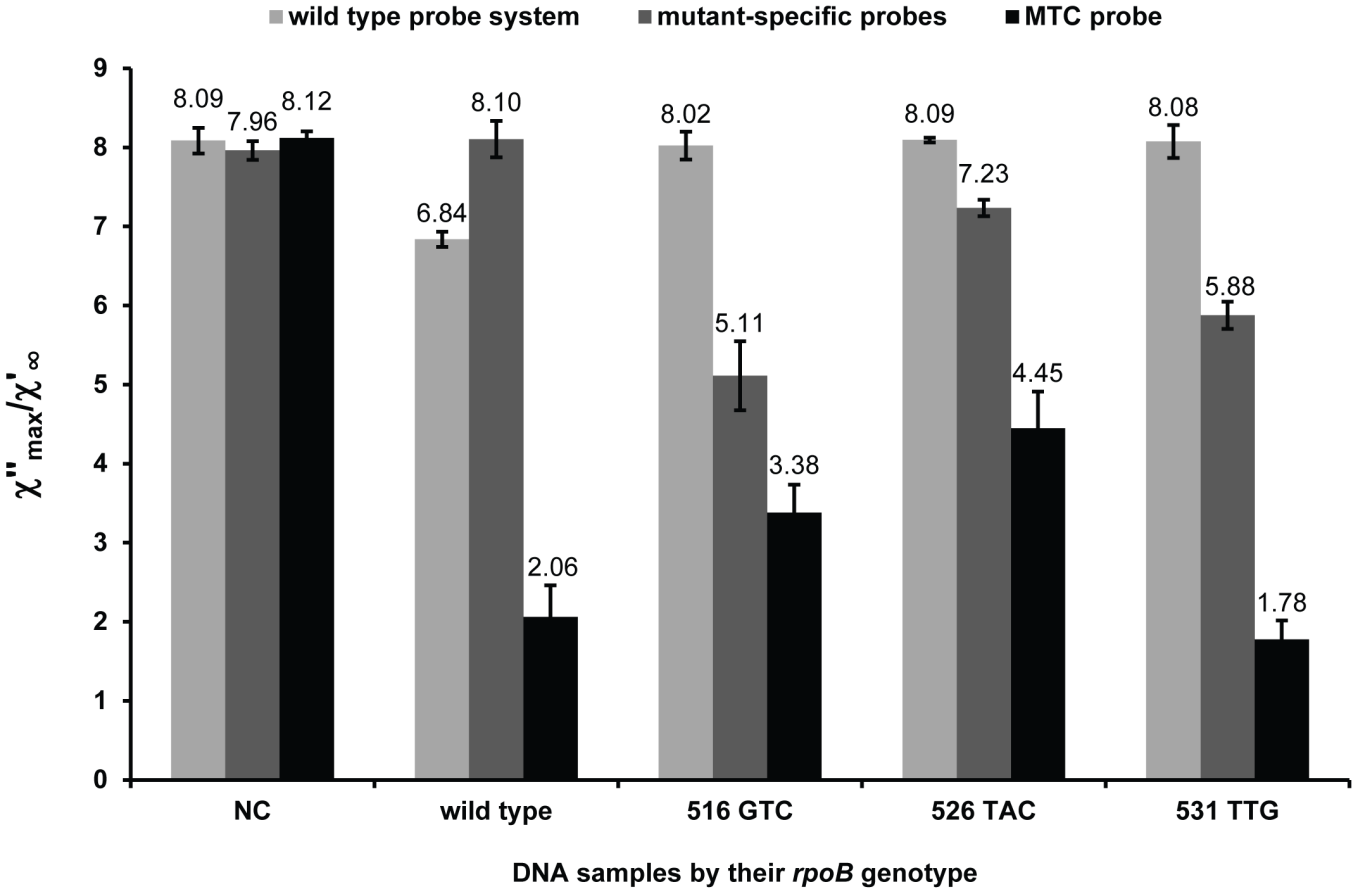
Complete assay tested on *M. tuberculosis* DNA samples. Complete assay (wild type probe system, cocktail of nine mutant-specific padlock probes, *M. tuberculosis* complex (MTC) padlock probe, and negative controls for respective probe or probe mix (NC)) was evaluated on four DNA samples from the RIF-susceptible reference strain *M. tuberculosis* H37Rv (wild type *rpoB*) and three RIF-resistant *M. tuberculosis* clinical isolates harboring a 516 GTC, 526 TAC or a 531 TTG *rpoB* mutation by the magnetic nanobead-based readout. To account for differences in iron-oxide content between samples, the 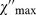 data were normalized using the constant value of the in-phase component of the volume susceptibility, 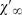.

LOD of the magnetic readout was determined with the wild type probe system on synthetic target representing a wild type RRDR. The LOD was determined to 10 amol target molecules (Figure S2). The complete assay was evaluated on four DNA samples; the RIF-susceptible reference strain *M. tuberculosis* H37R (wild type RRDR), and three RIF-resistant *M. tuberculosis* clinical isolates harboring an *rpoB* 516 GTC, 526 TAC or 531 TTG mutation. The assay successfully identified all four samples; the presence of *M. tuberculosis* complex DNA with, either a wild type RRDR, or a mutant version of the locus (Figure 5), without yielding false-positive signal from a mismatching probe, when compared to the signals of the negative controls. The 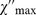 values were obtained from the complex susceptibility curves displayed in Figures S3–S4 in the supporting information.

## Discussion

We have developed a molecular method for detection of RIF resistance in *M. tuberculosis* by padlock probes and a magnetic bead-based readout (Figure 1). Padlock probes were designed to target the wild type RRDR region of *rpoB*, and the most frequent mutations giving rise to RIF resistance, i.e. at the first and second nucleotide positions of codons 516 and 526, and at the second nucleotide position of codon 531. A padlock probe for detection of the *M. tuberculosis* complex targeting the 16S–23S ITS region was also included in the assay. Ligated padlock probes were amplified by RCA and the VAM-NDA was used as a readout format.

The efficiency of the wild type probe system was improved by shortening the padlock probe arms, prolonging the backbone, and introducing a deletion and two mismatches in the longest gap-fill oligonucleotide. The great effect of this relatively slight change of the proportion of single-stranded and double-stranded parts of the DNA circle suggests that the original probe system was on the verge of not being able to bend and circularize at all. As the wild type probe system is still not as efficient as the single padlock probes, additional spacer nucleotides could probably increase the efficiency further. This example of an oligonucleotide assembly demonstrates the importance of accounting for tension in the duplex structure of circularizing or folding DNA probes. The new type of padlock probe system that we describe here could be used to investigate DNA sequences of other species and in other contexts where multiple variable bases are closely located in the target sequence.

The wild type probe system was shown to be specific for wild type target and to discriminate against all nine *rpoB* single nucleotide variants investigated (Figure 2). This strongly indicates that not only a single nucleotide mismatch at the very 3′ end of the padlock probe, but also that a mismatch at the position adjacent to the 3′ end prevents ligation. This was demonstrated by the lack of signal for *rpoB* targets harboring a 516 GTC, 526 CGC or 526 CTC mutation. In conclusion, the wild type padlock probe system will only generate a signal if all positions are wild type and hence, lack of signal demonstrates loss of wild type at any of the three codons. In the very rare occasion where a mutation is present at the first or third nucleotide position of codon 531, and at the third nucleotide position of codons 516 and 526 [5], it is likely that ligation would be greatly hindered and thus not yield a signal either.

Initially, all nine mutant-specific padlock probes were designed to target the same DNA strand, and despite efficient hybridization and ligation, it could be shown that the signal from the 516 GTC probe was greatly reduced when tested in the multiplex assay (Figure S5). We hypothesized that this reduction was due to blockage of target-primed RCA initiation by hybridization of downstream padlock probes targeting codons 526 and 531. Padlock probes designed for codons 516 and 531 do not compete for the same target sequencing binding site, but may block the 5′–3′ exonucleolytic digestion by phi29 polymerase that is required to acquire a free 3′ end adjacent to the ligated padlock for target-priming of the RCA process. In order to circumvent this problem during the multiplexed assay, the mutant-specific padlock probes targeting codon 516 were designed to target the coding DNA strand while the probes targeting codon 526 and 531 were designed to target the non-coding DNA strand.

The specificity and multiplexability of the nine mutant-specific padlock probes were evaluated on DNA extracted from eight RIF-resistant *M. tuberculosis* clinical isolates (harboring a mutation in either codon 516, 526 or 531), and on a synthetic target (representing the *rpoB* 526 GAC mutation) (Figure 3). All probes were shown to be specific for discrimination between their respective single nucleotide variant and the wild type sequence. In addition, the number of RCA products was not considerably affected when the reactions were performed nine-plex compared to single-, du-, or triplex (Figure 3). Notably, padlock probes designed for codon 526 and 531 seem to compete with each other for hybridization rather than blocking each other. This shows that the assay is highly flexible and multiplexable. For further extension of the method, more mutation-specific padlock probes could be added to the system. Ideally, a molecular method designed to detect mutations associated with drug resistance in *M. tuberculosis* should be both flexible and multiplexable in order to act in accordance with the continuously gained understanding of *M. tuberculosis* drug resistance and to address the different needs of assay design in various global settings.

During the probe design phase it was occasionally observed that hybridization, and subsequent ligation, of some probe versions were less efficient than others (as determined by the SMD method). The *M. tuberculosis* genome contains 65% GC [29], and the RRDR itself is known to form complicated secondary structures in form of hairpins [35]. It can be hypothesized that some of the original probes were not efficient due to secondary structure formation, probably both in the probe itself and in the single stranded DNA target. PCR-based methods have the advantage that the primers can be moved to a more AT-rich region for more efficient hybridization; however, due to the specific approach of single nucleotide variant detection used in this study, the probes could not be moved. Probe hybridization and ligation was improved for suboptimally performing probes by varying probe arm length, until satisfactory performance was achieved (Figure S6).

Evaluation of LOD for the magnetic readout format was performed with the wild type probe system since it yielded the lowest signal as determined by the SMD method (Figure 2) compared to the mutant-specific padlock probes (Figure 3). This is conceivable, since the wild type probe system requires hybridization of several oligonucleotides and three ligation events. The complete assay with the VAM-NDA as readout format was evaluated on four *M. tuberculosis* DNA samples. One was obtained from a RIF-susceptible strain and contains a wild type RRDR, while three were obtained from RIF-resistant strains, each harboring the most prevalent mutation in each of the investigated codons, i.e.: 516 GTC, 526 TAC and 531 TTG. All four samples were correctly identified by the assay as *M. tuberculosis* complex DNA, with either a wild type or a mutant version of RRDR (Figure 5). The wild type probe system yielded only signal in presence of wild type RRDR, and in the reversed scenario, the mutant-specific padlock probe cocktail yielded only signal in presence of a mutant version of RRDR. The assay was not only able to identify the most prevalent mutations in *rpoB* responsible for RIF-resistance in *M. tuberculosis*, but also confirmed loss of wild type and detected *M. tuberculosis* complex DNA. A mutation in the RRDR elsewhere than at the nucleotide positions investigated (*e.g.,* a silent mutation) can be expected to affect both the wild type probe system and the mutant-specific padlock probes, leading to loss of both signals. This generates a distinct signal profile from true negatives and positives, indicating the need for further analysis of the exact genotype. The 526 TAC mutant DNA sample yielded a significantly positive but slightly weak signal in the mutant-specific probe cocktail. SMD control measurements showed that this sample yielded an equal number of RCA products as the 516 GTC and 531 TTG mutant DNA samples (data not shown). We speculate that the particular padlock probe sequence (526 TAC) lead to a secondary structure formation which impeded hybridization of the oligonucleotide-conjugated magnetic nanobeads to the RCA products. The hybridization to the magnetic nanobeads can further be improved by adjusting hybridization conditions or by changing padlock probe backbone sequences.

Other readout formats in addition to the VAM-NDA could be explored for this padlock probe assay. For example, the SMD method used here to evaluate the probes has better sensitivity, although it requires more sophisticated equipment in its current form [33]. A colorimetric readout format, such as visualization by horse radish peroxidase and 3,3',5,5'-Tetramethylbenzidine, enabling parallel readout in a microplate, is another possibility [36]. The current LOD of the method developed here may be considered unsatisfactory for direct testing of clinical specimens. The sensitivity of the assay developed by Ke *et al.* [36] was increased by adding another cycle of circle-to-circle amplification, enabling a limit of detection of 600 targets per reaction. A similar increase in sensitivity can be expected for this assay by introducing an additional cycle of amplification to the current protocol. Furthermore, the assay in its current design is not able to provide information about which exact mutation is present in the target. If desired, this can be achieved by employing a multiplexed array readout. The assay could then also be expanded to detect mutations in other genes associated with drug resistance in *M. tuberculosis*.

Drug-resistant *M. tuberculosis* is a serious public health problem that threatens progress made in TB care and control worldwide. Drug resistance must not be ignored. Sharpened combined and novel efforts by the international community are needed to develop strategies to prevent further development of resistance, and to stop transmission of already drug-resistant strains. Molecular diagnostics can play an important part of future TB control. We have developed a molecular method for detection of RIF resistance in *M. tuberculosis* by padlock probes and a magnetic bead-based readout. We show that padlock probes can specifically detect mutations in the *rpoB* gene, and contrary to other PCR-based mutation detection methods [37], [38], [39], the assay is easily multiplexed and flexible.

## Supporting Information

Figure S1 **Confirmation of wild type probe system improvements.** Ten attomole synthetic DNA was used as template. The different padlock probe systems had the following properties. (A): Long target-hybridizing duplex part compared to the single-stranded backbone. (B): Shortened duplex and increased backbone. (C): The longest gap-fill oligonucleotide equipped with a “hinge”. (D): B and C combined. (NC): negative control for system D. RCA products were hybridized to fluorescent dye-coupled oligonucleotide and visualized in a confocal microscope.
(TIF)

Figure S2 **Determination of wild type probe system limit of detection by the magnetic bead-based readout format.** Ten-fold dilutions of synthetic target representing wild type rpoB locus was tested. Brownian relaxation frequency was measured for each sample and to account for differences in iron-oxide content between samples, the data were normalized using the constant value of the in-phase component of the volume susceptibility,. NC: negative control.
(TIF)

Figure S3 **Raw data of the experiment presented in Figure S2.**
(TIF)

Figure S4 **Full frequency scan data used to determine the normalized for each measurement presented in** **Figure 5** **.** Panels A–C displays data for the wild type probe system, cocktail of nine mutant-specific padlock probes, and M. tuberculosis complex padlock probe respectively while panel D displays the data for all negative control measurements.
(TIF)

Figure S5 **Investigation of blocking effects during RCA.** Padlock probes designed for the same DNA strand tested in singleplex (516 GTC) or duplex (531 TKG – degenerated probe at the 3′-end nucleotide of padlock probe) on M. tuberculosis clinical isolates harboring either a 516 GTC or 531 TTG mutation in rpoB, and compared with nine-plex assay in presence of all mutant specific padlock probes. RCA products were hybridized to fluorescent dye-coupled oligonucleotide and visualized in a confocal microscope. NC: negative control.
(TIF)

Figure S6 **Optimization of suboptimally performing padlock probes.** The target-hybridizing arms of the original degenerated padlock probes P5002, (rpoB 526 CKC) were shortened to yield P5926, which performed better on both 526 CTC and 526 CGC targets.
(TIF)

Table S1 **Oligonucleotides used in the study.**
(PDF)
