# Supplementary figures and images for: Detection of Rifampicin Resistance in Mycobacterium tuberculosis by Padlock Probes and Magnetic Nanobead-Based Readout
Source: PLoS One. 2013 Apr 22;8(4):e62015. doi: 10.1371/journal.pone.0062015 (PMC3632517; doi:10.1371/journal.pone.0062015)

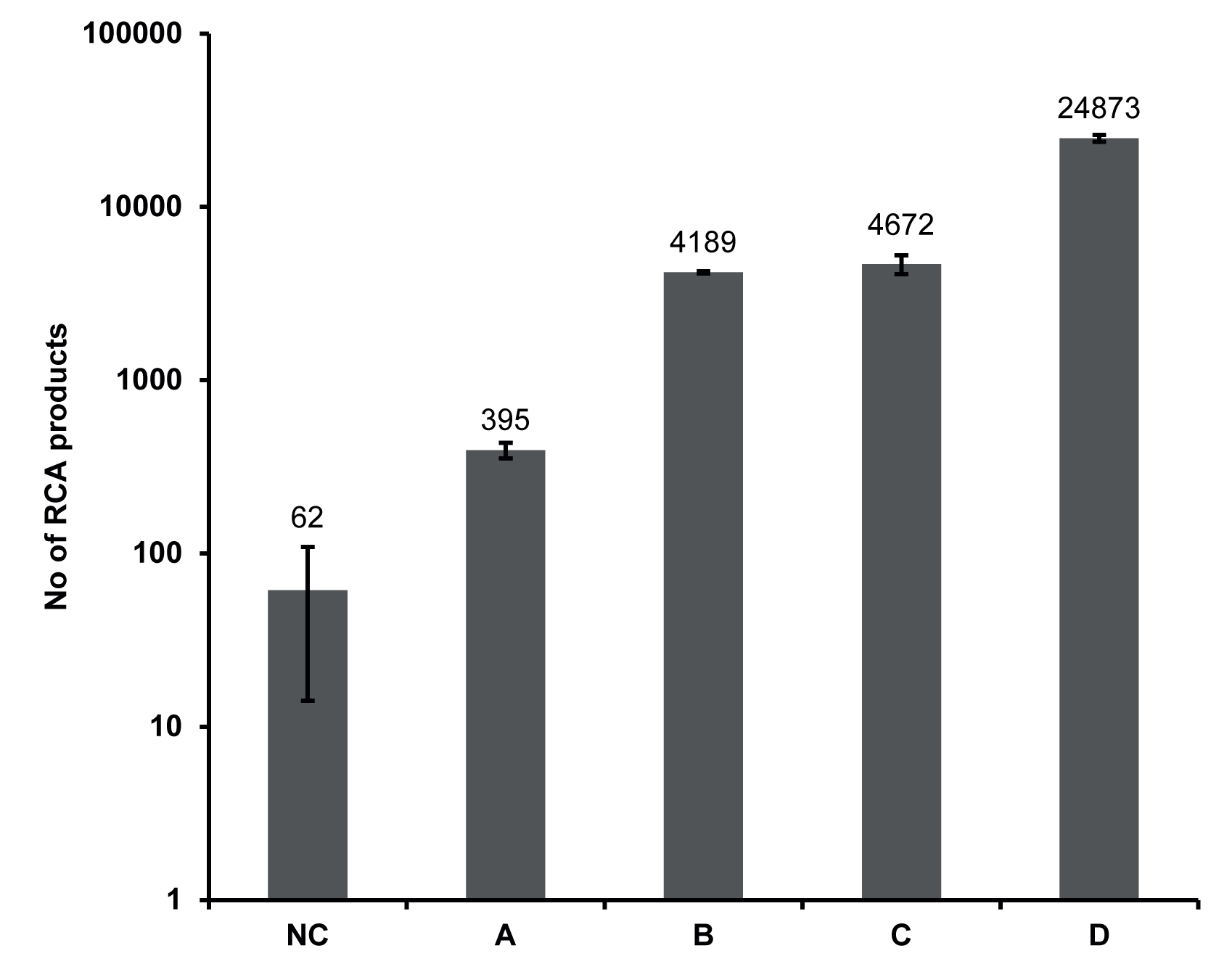

Supplement: Figure S1 — Confirmation of wild type probe system improvements. Ten attomole synthetic DNA was used as template. The different padlock probe systems had the following properties. (A): Long target-hybridizing duplex part compared to the single-stranded backbone. (B): Shortened duplex and increased backbone. (C): The longest gap-fill oligonucleotide equipped with a “hinge”. (D): B and C combined. (NC): negative control for system D. RCA products were hybridized to fluorescent dye-coupled oligonucleotide and visualized in a confocal microscope. (TIF) [file pone.0062015.s001.tif]

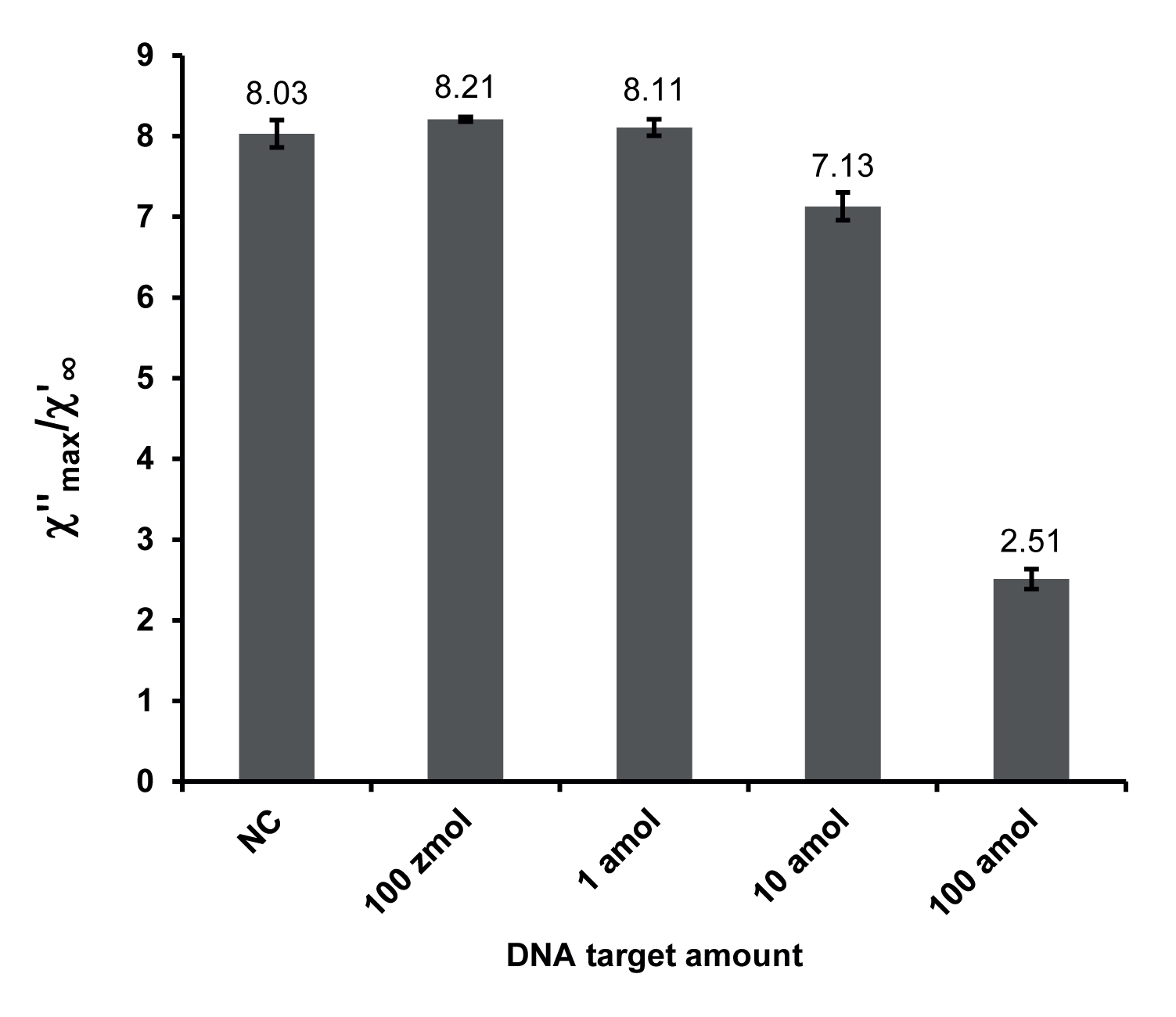

Supplement: Figure S2 — Determination of wild type probe system limit of detection by the magnetic bead-based readout format. Ten-fold dilutions of synthetic target representing wild type rpoB locus was tested. Brownian relaxation frequency was measured for each sample and to account for differences in iron-oxide content between samples, the data were normalized using the constant value of the in-phase component of the volume susceptibility,. NC: negative control. (TIF) [file pone.0062015.s002.tif]

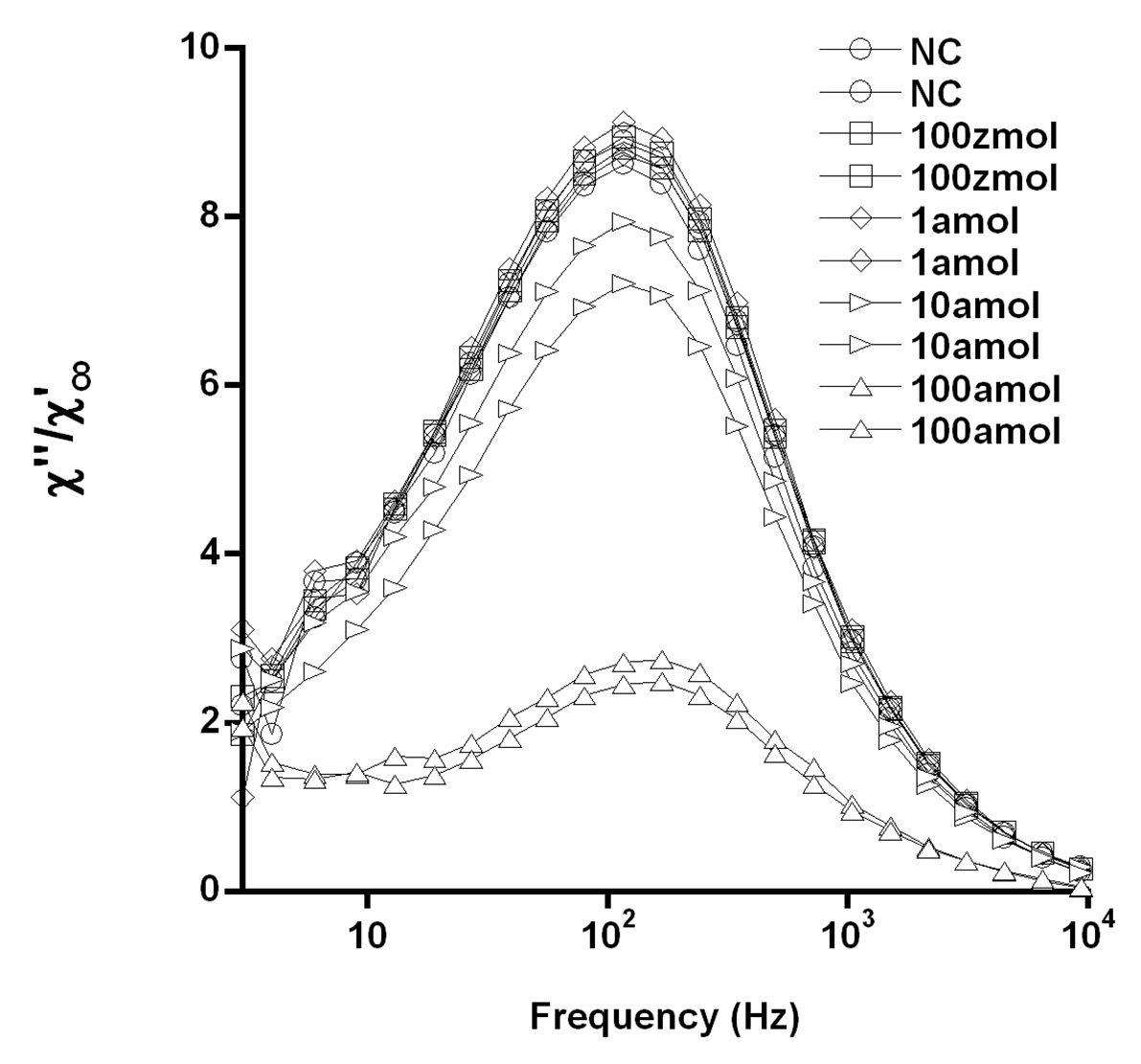

Supplement: Figure S3 — Raw data of the experiment presented in Figure S2. (TIF) [file pone.0062015.s003.tif]

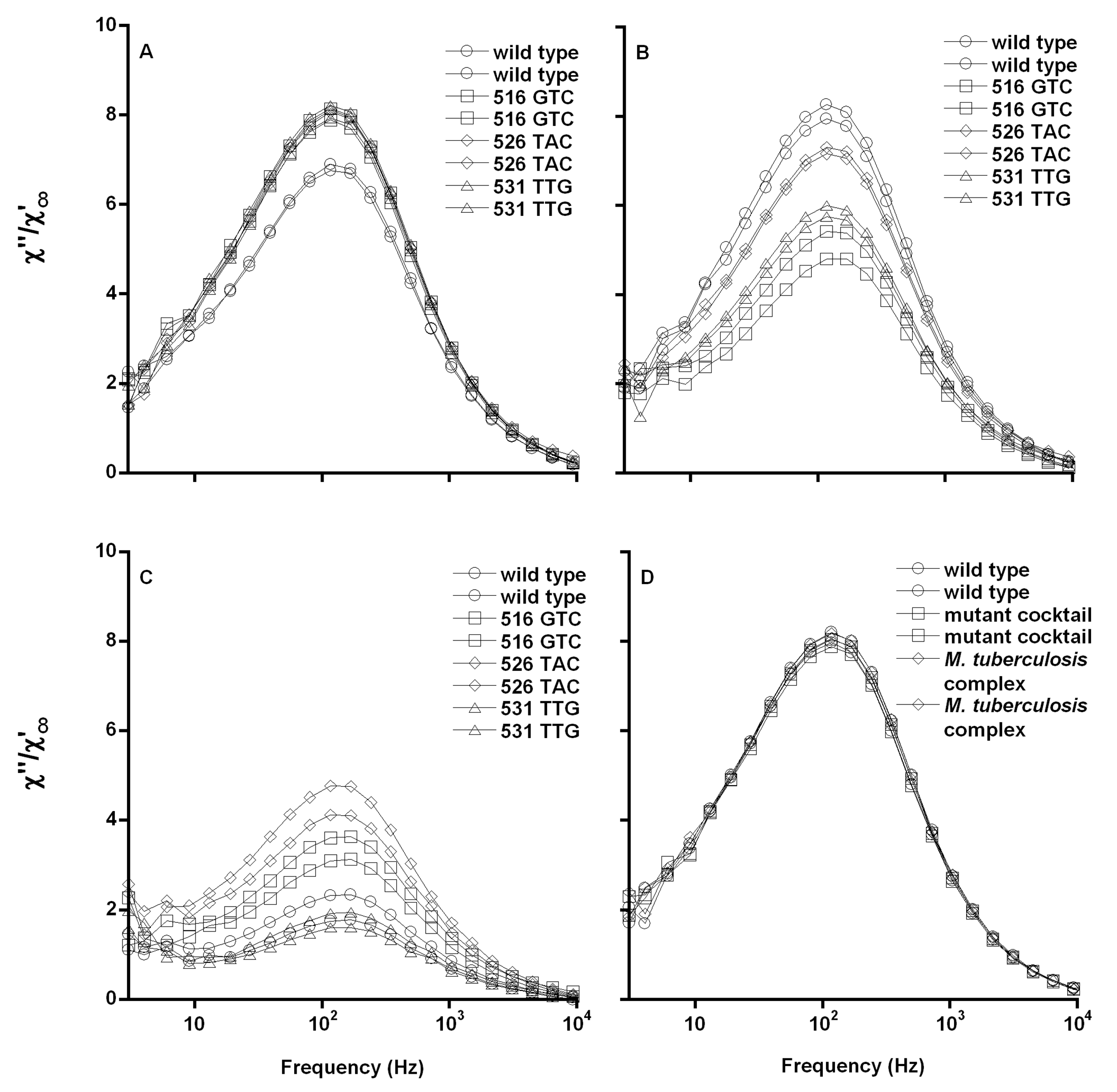

Supplement: Figure S4 — Full frequency scan data used to determine the normalized for each measurement presented in Figure 5 . Panels A–C displays data for the wild type probe system, cocktail of nine mutant-specific padlock probes, and M. tuberculosis complex padlock probe respectively while panel D displays the data for all negative control measurements. (TIF) [file pone.0062015.s004.tif]

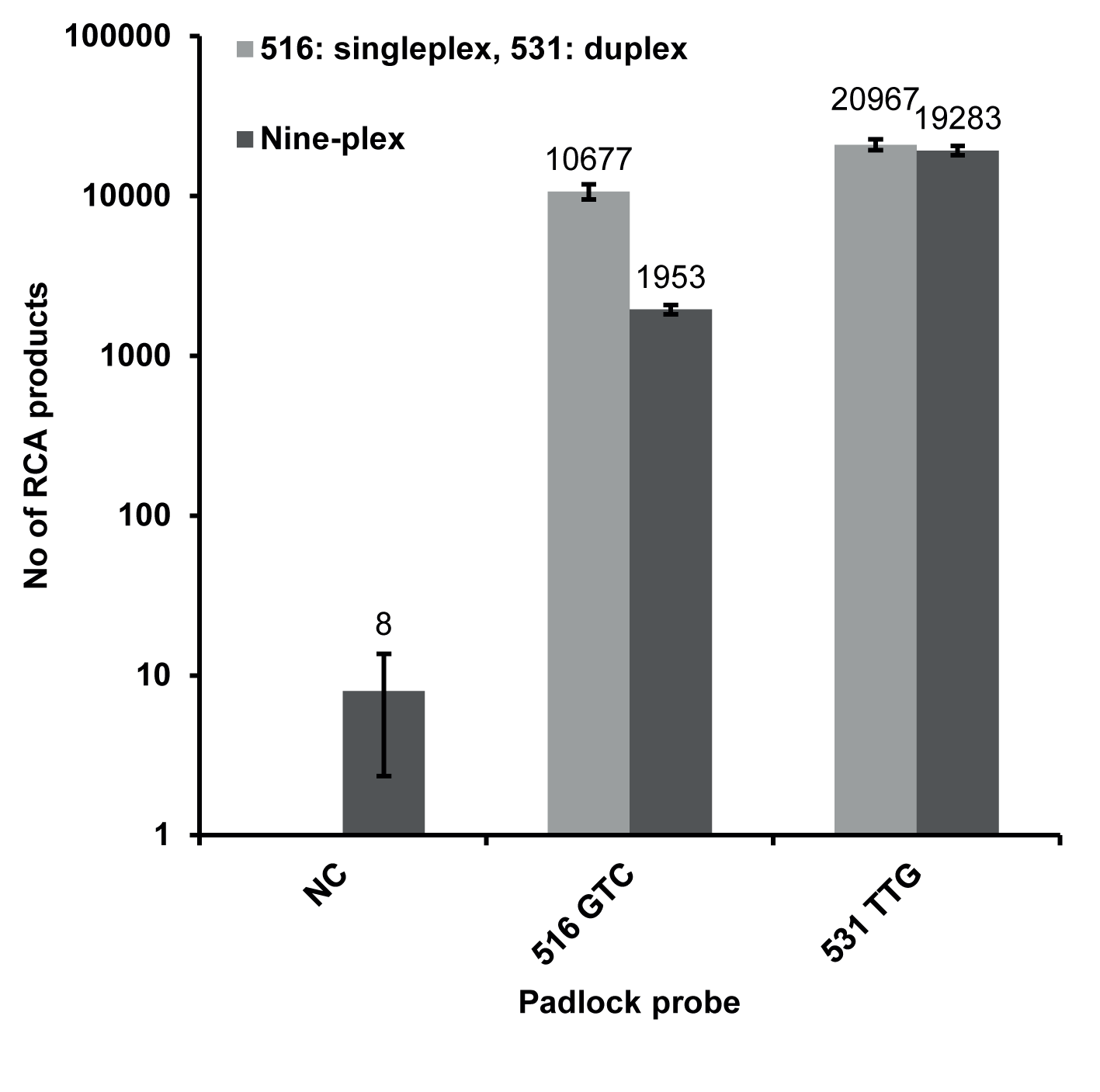

Supplement: Figure S5 — Investigation of blocking effects during RCA. Padlock probes designed for the same DNA strand tested in singleplex (516 GTC) or duplex (531 TKG – degenerated probe at the 3′-end nucleotide of padlock probe) on M. tuberculosis clinical isolates harboring either a 516 GTC or 531 TTG mutation in rpoB, and compared with nine-plex assay in presence of all mutant specific padlock probes. RCA products were hybridized to fluorescent dye-coupled oligonucleotide and visualized in a confocal microscope. NC: negative control. (TIF) [file pone.0062015.s005.tif]

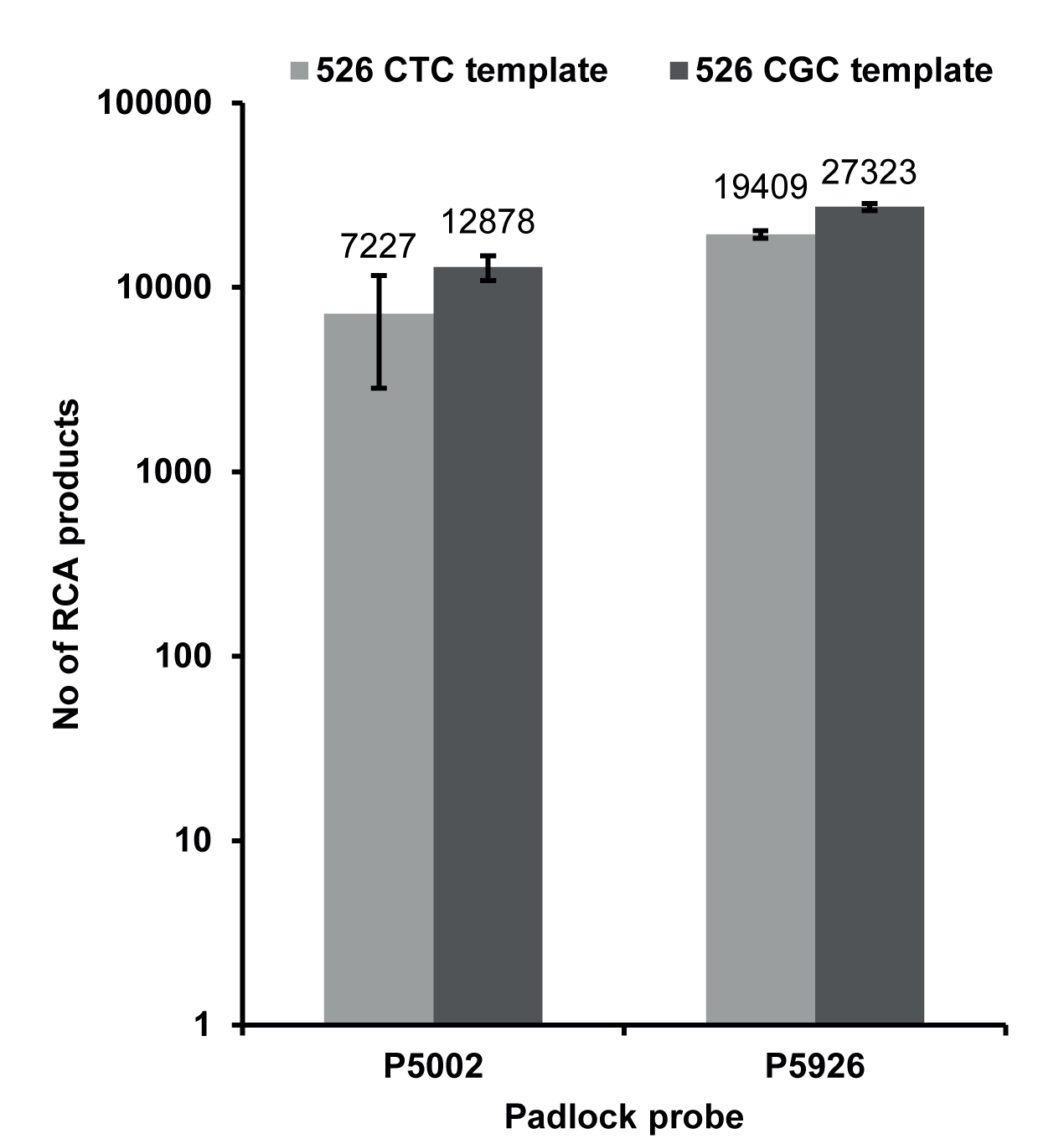

Supplement: Figure S6 — Optimization of suboptimally performing padlock probes. The target-hybridizing arms of the original degenerated padlock probes P5002, (rpoB 526 CKC) were shortened to yield P5926, which performed better on both 526 CTC and 526 CGC targets. (TIF) [file pone.0062015.s006.tif]
