# Supplementary material for: Detection of Rifampicin Resistance in Mycobacterium tuberculosis by Padlock Probes and Magnetic Nanobead-Based Readout
Source: PLoS One. 2013 Apr 22;8(4):e62015. doi: 10.1371/journal.pone.0062015 (PMC3632517; doi:10.1371/journal.pone.0062015)
Supplement: Table S1 — Oligonucleotides used in the study. (PDF) [file pone.0062015.s007.pdf]

Table S1. Padlock probes and oligonucleotides used in the study.

ID	Name	5'-3' sequence	Type of oligonucleotide	Modification
P4782	TB rpoB gf 516-531 wt v1	CATGAATTGGCTCAGCTGGGTGGATAGTGTCTTACACGAGTATGCAGCTCCTCAGTACGGGCCCCAGCGCCG	Padlock probe	
P4949	TB rpoB gf 516-531 wt v2	CATGAATTGGCTCAGTTTTTGTGGATAGTGTCTTACACGAGTATGCAGCTCCTCAGTATTTTTCCCCAGCGCCG	Padlock probe	
P5875	TB rpoB gf 516-531 wt v3	CATGAATTGGCTCAGTTTTTGTGCGACACATGACATCAACGTGTATGCAGCTCCTCAGTATTTTTCCCCAGCGCCG	Padlock probe	
L11170	TB rpoB gf 531-526 wt	ACAGTCGGCGCTTGTG	Gapfill oligo	
L11171	TB rpoB gf 526-516 wt	GGTCAACCCCGACAGCGGGTTGTTCTGGTC	Gapfill oligo	
L11420	TB rpoB gf 526-516 wt hinge	GGTCAACCCCGACAGCTCTTGTTCTGGTC	Gapfill oligo	
P5924	MND rpoB 526 DAC	GGTCAACCCCGACAGCGGGTGCGACACATGACATCAACGTGTATGCAGCTCCTCAGTACGACAGGCGGCGCTTGTH	Padlock probe	
P5925	MND rpoB 531 TKG	ACAGTCGGCGCTTGTGTGCGACACATGACATCAACGTGTATGCAGCTCCTCAGTAGCCCCAGCGCCM	Padlock probe	
P5926	MND rpoB 526 CKC	GGGTCAACCCCGACAGTGCGACACATGACATCAACGTGTATGCAGCTCCTCAGTAGACAGTCGGCGCTTGM	Padlock probe	
P5002	MNP rpoB 526 CKC	GGGTCAACCCCGACAGCGGGTGCGACACATGACATCAACGTGTATGCAGCTCCTCAGTACGACAGGCGGCGCTTGM	Padlock probe	
P5964	MND 516 TAC RS v1	ACCAGAACAACCCGCGTGCGACACATGACATCAACGTGTATGCAGCTCCTCAGTATGAGCCAATTCATGT	Padlock probe	
P5965	MND 516 GTC RS v1	CCAGAACAACCCGCTGGTGCGACACATGACATCAACGTGTATGCAGCTCCTCAGTACAGCTGAGCCAATTCATGGT	Padlock probe	
P5546	MND TB 16S-23S intergen species detection	CACCTGGAACAAGTCCGAGTGCGACACATGACATCAACGTGTATGCAGCTCCTCAGTAACCAAGGCGGTGGGACAA	Padlock probe	
L10919	TB rpoB wt SW	ATCACACCGCAGACGTTGATCAACATCCGGCCGGTGGTCGCCGCGATCAAGGAGTTCTTCGGCACCAGCCAGCTGAGCCAATTCAT GGACCAGAACAACCCGCTGTCGGGGTTGACCCACAAGCGCCGACTGTCGGCGCTGGGGCCCGGCGGTCTGTCACGT	Target	
L11485	TB rpoB 526 GAC	ATCACACCGCAGACGTTGATCAACATCCGGCCGGTGGTCGTCTCTCCCGCTGTCGGGGTTGACCGACAAGCGCCGACTGTCG	Target	
S00020	MND 516 RS CO v1	CTCTCTCTCTCTCTCTCTCTCTACGTGAGCGTGCCGGGCTGGAGGTCCGCGACGT	Capture oligo	5' Biotin
L10918	TB rpoB CO	CTCTCTCTCTCTCTCTCTCTCGACCACCGGCCGGATGTTGATCAACGTCTGCGGTGTGAT	Capture oligo	5' Biotin
L12890	BNL_RO_AluI	GTGTATGCAGCTCCTCAGTA	Restriction oligo	
L13179	1216 zip	GTTGATGTCATGTGTCGCAC	Detection oligo	5' Alexa Fluor 555
L10806	BNL_DO_AZ-	TCGTGTAAGACACTATCCACUUUU	Detection oligo	5' Cy3
L9261	1216 zip biotin	GTGTGTGTGTGTGTGTGTGTGTTGATGTCATGTGTCGCAC	Detection oligo	5' Biotin
